# A qualitative study of older adults’ perspectives on initiating exercise and mindfulness practice

**DOI:** 10.1186/s12877-019-1375-9

**Published:** 2019-12-23

**Authors:** Diana C. Parra, Julie Loebach Wetherell, Alexandria Van Zandt, Ross C. Brownson, Janardan Abhishek, Eric J. Lenze

**Affiliations:** 1 Program in Physical Therapy, Washington University in St. Louis, School of Medicine, 4444 Forest Park Ave, Campus Box 8502, St. Louis, MO 63108 USA; 2 VA San Diego Healthcare System and Department of Psychiatry, University of California, San Diego, 3350 La Jolla Village Drive San Diego, San Diego, CA 92161 USA; 3 Prevention Research Center in St. Louis, Brown School at Washington University in St. Louis, 1 Brookings Drive, Campus Box 1196, St. Louis, MO 63130 USA; 4 Department of Surgery (Division of Public Health Sciences), 660 S. Euclid Ave, Campus Box 8100, St. Louis, MO 63110 USA; 5 Alvin J. Siteman Cancer Center, Washington University School of Medicine, Washington University in St. Louis, 4921 Parkview Place, Saint Louis, MO USA; 6 Department of Biology, Washington University School of Medicine, Washington University in St. Louis, 1 Brookings Drive, St. Louis, MO 63130 USA; 7 Department of Psychiatry, Healthy Mind Lab., 600 S. Taylor Ave., St. Louis, MO 63110 USA

**Keywords:** Mindfulness-based stress reduction, Mindfulness, Exercise, Older adults, Qualitative study

## Abstract

**Background:**

Mindfulness practice and exercise are ways by which older adults can improve and maintain their physical, emotional and cognitive health.

**Methods:**

This single-site qualitative study gathered insights of older adults’ perceptions about initiating and maintaining mindfulness and exercise practices. We carried out focus groups with 41 adults aged 65–85 who had recently initiated Mindfulness Based Stress Reduction (MBSR), structured exercise, or their combination as part of participation in a clinical trial. We used a semi-structured interview to ask them open-ended questions regarding the benefits, barriers and facilitators of participating in mindfulness and/or exercise interventions. The interview also included questions regarding translation of these practices into community settings as well as the long-term maintenance potential of these practices.

**Results:**

Older adults indicated that the mindfulness training increased their awareness and self-reflection and fostered a more self-accepting attitude. Furthermore, they improved their self-care habits and reported having better familial and social relationships. The main barrier for both the exercise and Mindfulness group was time management. The social benefits and sense of community were some of the primary motivators for older adults in the exercise and/or MBSR interventions. However, the research on how to motivate older adults to initiate healthy behavioral changes also needs to be answered. The benefits of exercise and MBSR are a motivation in and of themselves, as indicated by some of the participants.

**Conclusions:**

This study indicates that mindfulness training and exercise can serve as tools to cultivate important health lifestyle qualities among older adults, who are in the midst of mental, social, emotional and physical change. If it were not for the purpose of the research or the incentives provided by the research team, these older adults may have never started the healthy behavioral changes. From the responses, this may indicate that older adults may need more incentives to begin and maintain behavioral changes other than for their own health benefit.

## Background

The number of people over 60 years old will double by 2050 [4], and ensuring that the elderly remain socially engaged and have the skills necessary to live a healthy life poses a major societal challenge [28]. Large-scale public health interventions should target this age group to improve physical and mental quality of life outcomes. For instance, educating older adults on efficient practices and strategies may help them adopt and maintain healthy behaviors as they age [1]. Unfortunately, researchers rarely explore the preferences and motivations of older adults in initiating or maintaining health-promoting activities such as exercise and mindfulness [15].

Exercise and mindfulness are promising interventions to benefit older adults both cognitively and physically. Exercise helps prevent falls [22] delays disability [21] enhances cognitive functioning [3, 8], improves depression [19] and reverses metabolic diseases [14]. Mindfulness reduces stress and worry [17, 27], improves mental health [9], sleep [13], awareness, self-efficacy [25], cognitive functioning, psychological well-being, and reduces systemic inflammation [6, 11, 23]. The most common forms of structured exercise are aerobic classes, combined aerobic and strength training, and broader physical activity promotion.

Mindfulness based Stress Reduction (MBSR) therapy is based on mindfulness meditation, where one observes the moment without judgement [16]. Dr. Kabat-Zinn describes mindfulness as steady attention and “*Rather than restricting attention to one object, however, this approach emphasizes the detached observation, from one moment to the next, of a constantly changing field of objects…”* [16]. MBSR was originally developed for patients who had chronic pain and stress-related disorders and now has become one of the most common type of mindfulness classes [2].

Community-wide proliferation and education about these healthy behaviors could provide the elderly with an easy and dependable way to remain both physically and cognitively fit. The inclusion of mindfulness practices and exercise in community programs could serve as a stepping-stone to ensure that older adults are well integrated in society. Szanton et al. investigated the effects and perceptions of MBSR training to minority older adults and found that the participants benefited from the MBSR meditation techniques in coping with stress. Participants also expressed their appreciation for the social support groups they formed during the intervention [26]. This is just one example of how MBSR training can empower older adults to handle the stresses of life and growing older.

This qualitative study examined the benefits and barriers of initiating and maintaining mindfulness and exercise from the perspective of older adult participants. Our study is unique and original in that we compared the perspectives from older adults who were in interventions that included MBSR with exercise, MBSR only and exercise only, all of which are effective and feasible interventions that promote healthy behaviors in older adults. Other studies, such as Moss et al. [20] examined the perspectives of older adults in an MBSR program compared to a control group that did not receive an intervention. Franco et al. [12] provided a systematic review of qualitative studies on older adult’s perspectives on exercise, but did not provide a comparison. A qualitative analysis of participants from the MEDEX study will be an invaluable tool in advancing our understanding the benefits, barriers, facilitators and maintenance strategies for older adults, which will further enhance future public health interventions. We utilized the preexisting MEDEX study to assess the participant’s perception of these interventions and to examine how these practices could be translated into the community.

## Methods

### Parent randomized controlled trial (MEDEX)

Our study uses a subset of participants from the MEDEX study to form focus groups to make a qualitative analysis of their perception of MBSR and/or exercise. The MEDEX study sought to investigate the mechanisms responsible for the beneficial effects of mindfulness and exercise on cognition, and neurobiological and metabolic functioning. The MEDEX study used a 2 × 2 factorial design randomized controlled trial (NCT02665481), in which participants were randomized to initiate exercise only, MBSR only, exercise and MBSR, or a health education control condition Inclusion criteria included: age 65–84, self-reported cognitive complaints, sedentary lifestyle (defined as no current participation in structured exercise for the purpose of improving fitness, more than 1 day/week for 30 min or longer), not currently practicing meditation.

Exclusion criteria included: diagnosis of dementia, mild neurocognitive disorder, or other neurodegenerative disease, psychotic disorders or other unstable psychiatric conditions, serious medical conditions that would prohibit safe participation in the interventions (e.g., cardiovascular disease) or would interfere with assessments (e.g., ferromagnetic implants which would interfere with MRI), language, hearing, or visual impairment that would prevent participation, alcohol abuse or illicit drug use within 6 month, current participation in cognitive training programs, use of medications that would interfere with or confound the results of assessments (e.g., glucocorticoids or diabetes medications).

The MEDEX study completed recruitment for the RCT in January 2019 and randomized *n* = 585 participants (291 in St. Louis and 294 in San Diego). Participants mean age was 71.5 years old (SD = 4.8), 73% were women and 75% were non-Hispanic White. The MEDEX recruited participants primarily via media stories (both newspaper and television), online sources, targeted mailings, word of mouth, flyers, and research participant registries (Table 1). The MEDEX study was able to attain an adequate level of racial and ethnic diversity among the participants, while also maintaining the English-speaking requirement.

**Table 1:** Recruitment by source in the RCT of the MEDEX parent study (total *n* = 585 randomized)

Recruitment tactic	Number of randomized participants from this source	Percentage of sample from this source
Online sources (i.e., website, Facebook, Trialspark)	146	25.5%
Press (Television, Newspaper, Magazine, etc...)	145	25.3%
Recruitment Letter	89	15.5%
Word of Mouth	79	13.8%
Flyer	47	8.2%
Other/unknown	37	6.5%
University-wide research registry	21	3.7%
Referred from different study	8	1.4%
Physician Referral	1	0.2%

Each intervention lasted for 18 months and each initially began with structured group sessions led by professional instructors who were well-trained and highly experienced individuals. They were specifically taught to carry out structured instructional protocols focused on older adults. Instructors followed a manual to ensure consistency between the research sites (St. Louis and San Diego).

The exercise group met 2x/week for the first 6 months and then 1x/week for the remaining 12 months. An experienced group instructor led the sessions, instructing participants on aerobic, resistance and balance/mobility exercises. During the aerobic training, participants used treadmills, elliptical machines and/or stationary bikes to achieve 65% of their peak heart rate at baseline and then gradually progress to 70–85% of their peak heart rate. For strength training, participants were to perform one or two sets of 12–15 repetitions of 10 weightlifting exercises at 65% of their one-repetition maximum (1-RM) for the first 4–5 weeks. Participants then gradually increased to 2 sets of 8–10 repetitions at their 80% 1-RM. The functional resistance training included whole body compound muscle movements using bodyweight, dumbbells and resistance bands. Balance training included dynamic movements to challenge postural stability. The instructors encouraged participants to achieve an additional 150 min/week of focused aerobic and functional activities at home.

The standard MBSR protocol [24], as developed by Jon Kabat-Zinn, Ph.D. was used for the MBSR intervention. All instructors had at least 4 years of experience and a personal mindfulness practice. Participants underwent orientation and then met for eight 2.5-h classes and one four-hour silent retreat on site where they practiced mindful movement, mindful walking and sitting meditation. MBSR training included mindful breathing, eating, listening, and everyday mindfulness; sitting, walking, and lying meditation; mindful movement (yoga); and loving-kindness meditation. Daily home practice was also assigned to enhance their learning. After the eight-week class, participants were to attend instructor led monthly booster sessions assigned daily home practice up to 45 min per day of formal meditative activities for the remaining 16 months. The combination MBSR + exercise group followed the same schedule as the MBSR plus the same exercise schedule as the exercise alone group.

#### Adherence and Fidelity

The study provided participants with a smartphone or tablet to record their at-home practice. The survey asks how many minutes of exercise and/or MBSR they have completed that day; this serves as both a measurement and reinforcement of adherence.

To ensure consistency and fidelity, the instructors had regular phone calls to discuss exercise training techniques. MBSR and Health education instructors met weekly with a supervisor.

### Focus groups

We recruited participants for our study from the St. Louis site by sending out an email asking if they would be willing to participate in a focus group to discuss their perceptions of the study. Those who were willing to participate were asked to respond with dates and times they would be available to come. Participation in the focus groups was voluntary and consent was obtained prior to the beginning of each session. Light snacks and a gift card were provided to the participants for their involvement. The facilitator of the focus groups was not associated with the MEDEX study and was independent from the research team; this information was disclosed to participants before beginning the sessions, thus reducing the likelihood of obtaining biased answers. Written notes and video recordings were taken during the sessions. The video recordings were transcribed by a research assistant and checked for accuracy. No names or personal identifiers were kept on the transcripts. The average age was 70.7 years old, with a range of 65.1–81.8 years old. Table 2 includes the male to female ratio. The groups had an uneven distribution of males to females, with the MBSR group having more males and the other groups having more females. Overall, there were more females than males. Regarding race distribution, 75.6% of the participants were White, 19.5% were Black or African American, and 4.9% were more than one race.

**Table 2:** Male:Female ratio

Group	Total Number	Males	Females	Male:Female ratio
Exercise	8	2	6	0.33
MBSR	6	4	2	2
Exercise + MBSR	10	2	8	0.25
Exercise + MBSR	9	2	7	0.29
MBSR	8	2	6	0.33
Total	41	12	29	0.41

Five focus groups, comprised of two Mindfulness groups, two mindfulness and exercise groups, and one exercise only group, were completed during November 2017. This study combined those five groups into 3 groups: Mindfulness, Exercise and MBSR and Exercise. There were 41 total participants with ages ranging from 65 to 85 years old. 29% of the participants (*n* = 12) were males and 71% were females (*n* = 29). There were 14 participants from the Mindfulness groups, 19 from the combination MBSR + exercise group, and 8 from the exercise group. A semi-structured interview guide was used during the sessions (Additional file 1). Each group session lasted an average of 60 min. Participants were asked questions regarding the benefits of participating in the interventions, barriers and facilitators of participation, and ways to translate these interventions into community settings including self-management, technology delivered interventions, peer coach programs delivered by older adults, open gym alternatives, and supervised and guided programs or other community engagement opportunities.

Data were analyzed using NVivo 1 1[18]. Transcripts from the sessions were imported and coded according to group. Data-driven nodes were created, and transcripts were manually coded using deductive coding that was developed before examining the data. Codes included benefits, barriers, facilitators, maintenance, and community translation. Two independent reviewers coded the data, and discrepancies were discussed and solved until a rate of 90% agreement was obtained. If a disagreement still existed, a third reviewer coded the data based on the input from both reviewers. After the descriptive and thematic analysis, representative quotes for each theme and for each group were selected.

We selected 5 main questions and developed themes based on those responses. The main questions included benefits, barriers, facilitators, maintenance and community. The original questions in the interview are listed in Additional file 1. We took the responses that were directly answering those questions that pertained to those themes. We then analyzed those answers and developed themes based on the responses, and color-coded the themes. Answers could also have two themes if it was relevant for both themes. All of the raw data from the focus groups has been made available as Additional file 2: The data was then organized into a visual color-coded chart to show the percentage of each theme represented by the answers to the question.

The number of responses varied between the questions and between groups as the interview was non-structured and informal.

The benefits section includes the themes mental health, physical health and social health. The barriers section includes parking, staff, technology, feedback & frequency, intervention logistics, time management and motivation. Facilitators were broken into the intervention, staff and individual. Maintenance included community settings and individual practice. Finally, Community translation included community centers, parks and other pre-existing programs.

We added the visual graphs as pie charts after each of the main titles: benefits, barriers, facilitators, maintenance and community translation.

## Results

### Subject characteristics

A summary of the topics identified by each group according to the theme is provided below.

#### Benefits

Benefits was categorized into mental, physical and social benefits. Participants indicated many benefits of their participation in the mindfulness and exercise interventions, including increased awareness of thoughts, emotions, bodily sensations, and behavior during their daily lives. They also mentioned improvement in physical function and ability to perform activities of daily living. In addition, they placed emphasis on the importance of the trainers to help them correct and improve form from sports-related movements or simply activities of daily life.

##### Exercise group

Older adults perceived their exercise program as beneficial as it increased their mobility and strength. Others reported that learning the proper form was most useful for personal function and carrying out activities of daily living. Some seemed to appreciate feeling their muscles working and learning about their body, even if they were a little sore the following day.

*Example quotes:*

“I learned that having some exercises are beneficial for mobility. Items that help me get out of the chair, get off the floor, and move around.”

“The trainers were very helpful in making small corrections to our form. We have lived in these bodies for a long time. I learned that when I was doing things I was doing something a little wrong that made a difference. All of the sessions were helpful and both trying to correct and optimize our form and to help us to do activities of daily living.”

“I think all of us were introduced to new machines and exercise that maybe we have not done before and the next morning you would definitely feel it. It made you aware that those muscles were working. Maybe the soreness was a barrier. I was never that sore, it seemed that they always seemed to get the number right.”

##### Mindfulness group

Overall, older adults perceived mindfulness training to benefit their mental and emotional state of mind. Participants reported that the mindfulness training helped them be more aware of the present moment and to be less judgmental toward themselves. Several older adults reported being more relaxed, calm and understanding. Others reported having more discipline, self-encouragement and a greater ability to overcome obstacles. Decreased tension and anger, improved sleep and peace of mind were additional benefits perceived with mindfulness training.

*Example quotes:*

“More relaxed and being aware of what is around me. Looking at sights and sounds and what is around me…my receptors are working.”

“… lessens the tension and stress that I experience. I also like yoga exercise because I feel the benefits.”

“I have calmed down a lot; little things do not get to me anymore.”

“Very reassuring, I have been feeling terrified about what is going on with me. I thought I had Alzheimer’s, I didn’t know what to do. This has made me calm down and realize that there are many more things to focus on in life.”

“I am falling asleep so much better, my mind is not wandering anymore.”

##### Mindfulness and exercise group

In the mindfulness and exercise group, older adults reported feeling stronger, having better balance, flexibility and more confidence in walking without falling. Some claimed to have more energy and reduced back pain. Furthermore, participants revealed they felt less stress, self-hatred and more patient, self-confident and improved their outlook on life. Both exercise and mindfulness were perceived as beneficial, although, one participant felt that mindfulness was more important to learn.

*Example quotes:*

“… I think the mindfulness was more important to learn and retain than the exercise part of it because of changes in my outlook and what I am thinking. Physical changes throughout the years have decreased no matter what I do, but my mind can improve.”

“I don’t hate myself as much as I used to. I just let it go, it doesn’t matter… Now I can make a decision faster and I agree with what I am putting down. If you screw up, move on, it’s okay, just let it go.”

“I think a lot of it is self-confidence. Being confident of walking down the street and not falling down the stairs, or street. It is because I have made myself more aware of myself.”

#### Social benefits

##### Exercise group

Participants in the exercise only group felt that going to an exercise class was a valuable time to make new friends and form a community.

*Example quotes:*

“It is a social group, and the staff that helped make it a true community.”

“I think is just the friendships and relationships formed would be my primary benefit.”

##### Mindfulness group

The participants in the mindfulness training group perceived several social benefits including improved marriage relationships and being more connected with people in their group. As mentioned previously, participants reported feeling less anger, tension and frustration, which would translate into better relationships.

*Example quotes:*

“…significant change in my marriage relationship mainly from being aware of tension points and having alternatives ways to deal with such.”

##### Mindfulness and exercise group

The participants in the Mindfulness and Exercise group felt that their experience resulted in new relationships and enjoyed hanging out with members in the group. Furthermore, the group aspect helped keep individuals accountable. Some also had more self-confidence in their ability to walk without falling. They also reported enjoying the sense of community they formed with their group. Interestingly, several older adults who were retired reported missing the stress and structure of their jobs, and this opportunity of mindfulness and exercise training provided some of that needed structure.

*Example quotes:*

“I think the social part is huge, hanging out with people who are doing the same thing as you. Everyone needs acceptance and inclusion...”

“It has helped my family too… I have been able to be more in touch with my mind and feeling my body. I have an increased sense of awareness in the community. I can run a little bit, and because I also have awareness of my body in space…I am so much more in the now, less in planning and focusing on everything else.”

**Fig. 1:**
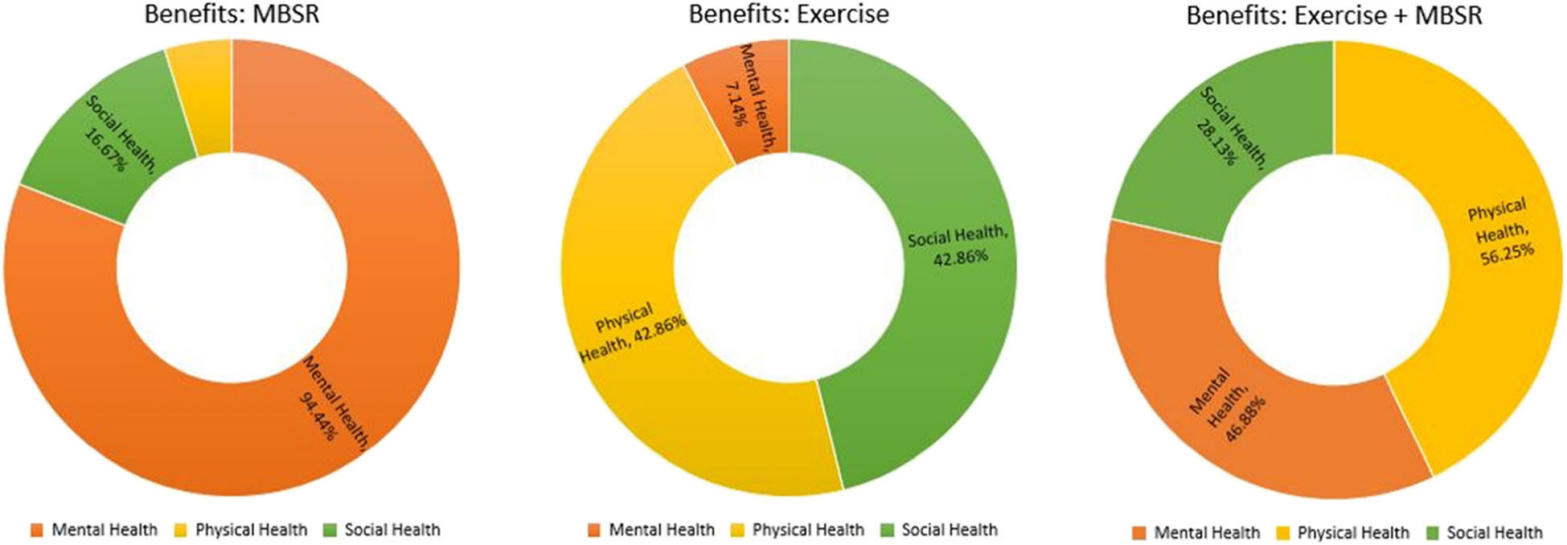
Comparing Benefits among Groups

Figure 1 depicts the visual comparison of the different benefits among groups. The MBSR group primarily experienced mental benefits, whereas the exercise group experienced mostly physical and social benefits. The combination group (exercise + MBSR) experienced a fairly equal distribution between the three benefits.

#### Barriers

Barriers for participation in the interventions were explored. Intervention barriers included staff, intervention logistics (e.g. false expectations, wishing to be a part of a different group, miscommunication), time management, feedback and frequency, parking and motivation. Several participants found it hard to practice mindfulness in their daily life due to prior commitments and scheduling conflicts in spite of being retired.

##### Exercise group

For the exercise group, participants felt that keeping up with the functional training at home was challenging because there was no feedback or structure. A few participants also mentioned it would have been beneficial to have more education on the exercises they were doing and why they were doing them. Some others mentioned that there was a lack of feedback from the trainers.

*Example quotes:*

“I have a very busy life, so trying to actually follow through and make time to practice what I agreed to participate to.”

“I didn’t realize how busy I was until I started to do this program.”

##### Mindfulness group

Several participants in the Mindfulness group stated they were already very busy and that it was difficult to follow through. Others mentioned interruptions at home were barriers in continuing the practice on their own.

*Example quotes:*

“I tried to do 45 minutes a day [of home practice], which I thought was not very much. But oh my gosh, that is a huge amount of time…”

##### Mindfulness and exercise group

There were mixed feelings of barriers in the mindfulness and exercise group. Some participants stated they were very busy and the training was too much of a time commitment. Others mentioned that they wanted more sessions for motivation and to keep them accountable. Some perceived the activities of mindfulness as being too individual (not group oriented or promoting social interaction), while others used the time as a social event. Barriers of time commitment is very personal and varies depending on the individual’s state in life.

*Example quotes:*

“I am still working, so coming 3 times a week was a real challenge. I missed the mindfulness going back to once a month [in the maintenance phase].”

**Fig. 2:**
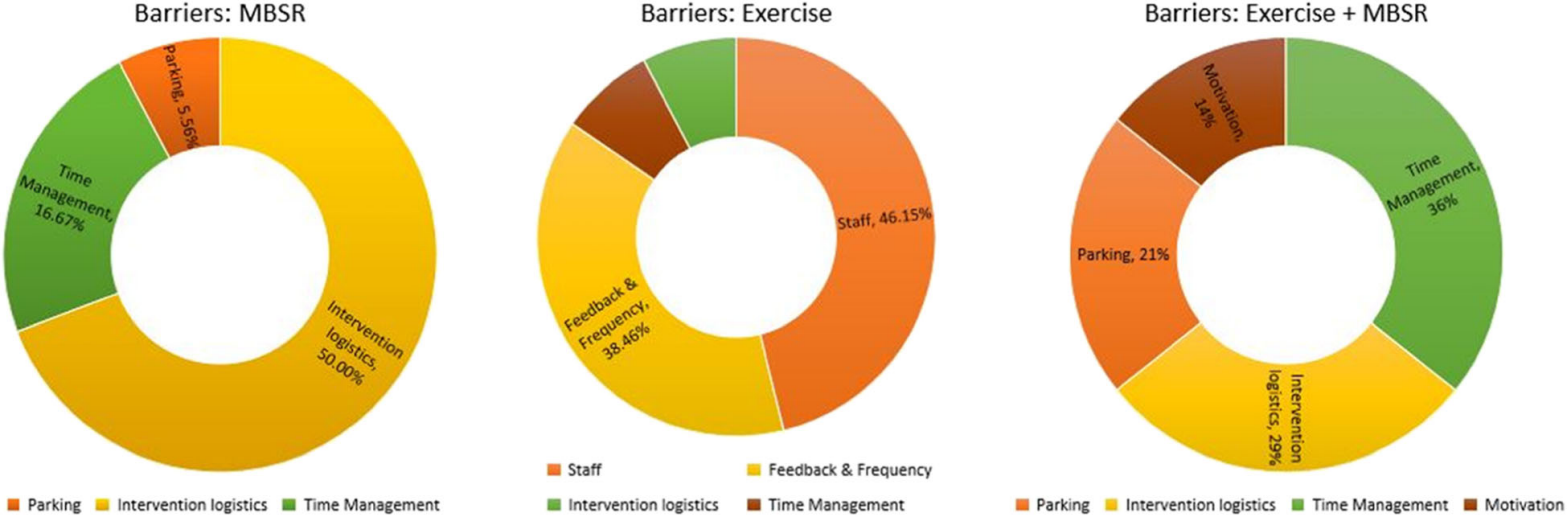
Comparing Barriers among Groups

Figure 2 shows the comparison of barriers across the groups, which includes staff, intervention logistics, feedback frequency, time management, parking and motivation. The primary barrier for the MBSR group was intervention logistics, which was mostly due to miscommunication, not receiving the medical results and desiring to be a part of the other group. The exercise group’s primary barriers include staff, as well as feedback and frequency of the sessions. The combination group (exercise + MBSR) had a balanced distribution of barriers, which includes parking, intervention logistics, time management and motivation.

#### Facilitators

Facilitators of the participation in the different intervention groups included the following categories, “intervention (e.g. stipends, accommodations), staff, and individual facilitators (e.g. personal benefits, goals)”.

##### Exercise group

Participants in the exercise group stated how much the support, encouragement and feedback from the trainers was important for their motivation and learning how to listen to their body. Others reported that the benefits of exercise, such as decreased blood pressure, improved energy and feeling stronger motivated them to exercise.

*Example quotes:*

“The trainers, the support, they were really helpful.”

“It was encouraging to see the results, I am stronger, and I felt better.”

##### Mindfulness group

The Mindfulness group stated that their relationship with each other and the staff. The participants highlighted how the staff facilitated their motivation for participating.

*Example quote:*

“I had a good relationship with a lot of them. They made it easier to come.”

##### Mindfulness and exercise group

The mindfulness and exercise group also reported the importance of staff influencing their motivation. The staff helped keep them accountable and positive reinforcement motivated individuals to do the training.

*Example quote:*

“The positive reinforcement was amazing. There was never too much, it felt nice to be congratulated and honored. “

**Fig. 3:**
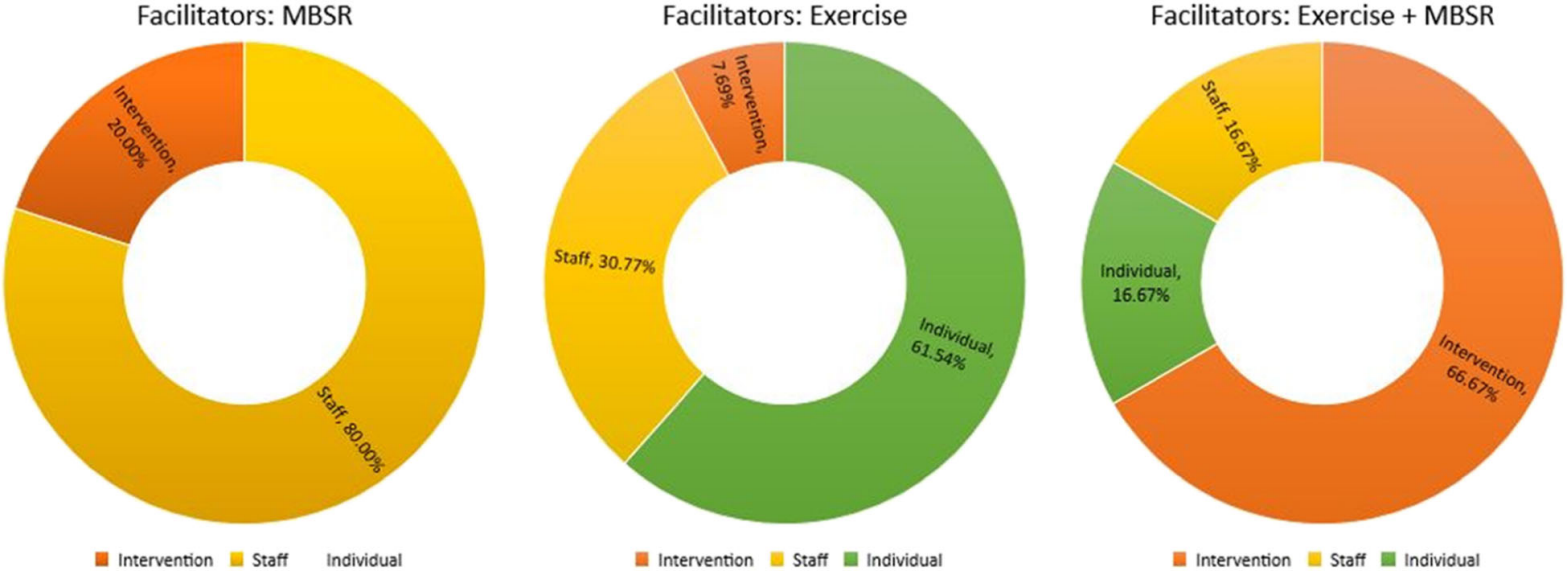
Comparing Facilitators among Groups

Figure 3 compares the facilitators among the 3 groups. The staff was the primary facilitator in the MBSR group, the individual for the exercise group and the intervention for the combination group (exercise and MBSR).

#### Maintenance

We asked participants about strategies that they could use to stay engaged and motivated to continue the practices learned during the study. Two subthemes were identified including “community settings” and “individual practice” which included motivation and strategies.

Below are selected quotes that relate to facilitators from the individual practice and the community settings. All of the participants except one said that they would be willing to extend their participation in the program. The participant who declined mentioned time constraints as the limitation.

##### Exercise group

The Exercise group related that feedback and positive reinforcement would be an important component in their maintenance. The majority of respondents were enthusiastic and motivated to continue with their exercises and all participants were willing to participate in them for the long term.

*Example quote:*

“I think the feedback portion would be critical or incentive or reward when turning in the weekly exercises.”

##### Mindfulness group

In the Mindfulness group, participants stated their perceived benefits motivates them to maintain their exercises. Others stated they would change the meditations every once in a while, so they would not get bored. One individual mentioned keeping the routine of doing yoga at the same time to help with maintenance.

*Example quotes:*

“I will continue to mediate because it helps me and I see the benefits in it. This study has given me tools to continue to practice for the rest of my life.”

“The thing that keeps me motivated is the mindful movement in the morning because it loosens up my joints, which is a motivation right there. I have my watch set to do a body scan, and hopefully do it every day. I feel better, I don’t think I need an external motivation.”

“I do the yoga and meditation at the same time every day, so it has become part of my routine in my daily schedule.”

##### Exercise and mindfulness group

Several participants stated they would continue to do their exercises, and mentioned the importance of goal setting and making the goals personal as a way to ensure they continue with their exercises. Another person mentioned making goals relevant to everyday life was a useful tool to ensure maintenance.

*Example quotes:*

“If the goals become personal, there is a reason to continue the exercise.”

“I am far more religious going to the gym and getting my exercises done since this study has ended.”

“The thing that keeps me motivated is the mindful movement in the morning because it loosens up my joints, which is a motivation right there. I have my watch set to do a body scan, and hopefully do it every day. I feel better, I don’t think I need an external motivation.”

#### Community translation

##### Exercise group

Programs for the elderly are already in place, for example, one of the participants stated they might continue with the silver sneakers program, a free fitness program for older adults.

*Example quotes:*

“The silver sneakers-once you have it you do not have to pay for it over and over again.”

“There are free dance programs some days of the week, and different things to do throughout the community that are exercise related.”

“We would be able to go outside and walk around all of the parks here.”

##### Mindfulness group

Most of the participants wanted to see this intervention in the community. A few options participants suggested were offering classes at libraries, community colleges, Silver Sneakers or OASIS international. Some others were hesitant in continuing mindfulness training with strangers.

*Example quotes:*

“Community centers and libraries would be good.”

“OASIS international, they do a lot of health and education training.”

##### Exercise and mindfulness group

The Exercise and Mindfulness Group expressed interest in having these interventions brought to the community. Some suggested having the city provide a program for the community or corporations implement this for their employees. Another person thought it should include people of all ages and not just seniors.

*Example quotes:*

“I would like to see corporations do it with their employees.”

“We are only focusing on seniors, but I think it could be dispersed throughout many different age groups.”

“On Saturdays during the summer, Tower Grove [a city park] has yoga there. I was wondering if the city could offer something like this throughout.”

## Discussion

This study indicated how older adults perceived mindfulness and exercise to be helpful in improving their mental, physical and social health. This is important for understanding how older adults might make positive behavior changes – mental, physical, and social – relatively late in life, and then maintain them over the long term.

Responses revealed that the benefits of exercise included enhanced mobility and stronger muscles. Participants stated that the primary benefits of mindfulness included reduced stress, better sleep, and positive changes in their perspective on life, improved relationships and a greater connection with the community. These results are consistent with the findings of Satter Moss et al. [20] and Franco et al .[12], who also used qualitative methods to investigate the effects of mindfulness and exercise. Our results are also consistent with Szanton et al. [26] who investigated MBSR only. To our knowledge, this is the first study to include perspectives from older adults who initiated and then maintained practice in mindfulness, exercise, and their combination. Overall, our findings suggest that the potential benefits of mindfulness training may encompass a broader range of domains than the benefits of exercise, including positive effects on family and social and marital functioning.

Time commitment was the most prominent barrier, as many participants stated that they are very busy despite being retired. Other barriers included interruptions at home and frequent travelling. Franco et al .[12] conducted a systematic review and found that older adults resist exercise due to competing priorities, social awkwardness and physical limitations. Another barrier to exercise commonly seen in older adults is fear of hurting oneself or falling [7] and one of the individuals interviewed revealed that they felt more confident after the training to walk on streets and not fear falling.

Another clear factor to emerge from the focus groups was the importance of facilitators in helping older adults maintain an exercise and meditation routine. In this study, facilitators for the participants included the exercise trainers and mindfulness instructors, who motivated participants by showing their care and concern for their well-being. Several factors could have contributed to this finding; first, the instructors were well-trained and highly experienced individuals. Second, they were specifically taught to carry out structured instructional protocols focused on older adults. This finding highlights the fact that these benefits may not be gained if classes are peer-led versus structured and professionally guided. The participants also stated how much they appreciated the trainers showing them new skills and how to exercise properly. Other studies have also highlighted the importance of having trainers and positive reinforcements for exercise motivation and adherence [5, 10]. Furthermore, several participants mentioned how they enjoyed the social aspect of the exercise groups. The social interaction and special supervision are important facilitators in helping older adults adopt and maintain exercise routines [10, 12]. Individual facilitators included aspects such as having a dog, telling friends to make them accountable, or marking it on their calendar.

Maintaining exercise or mindfulness practice is essential for continuing their benefits over the long term. Many of the participants also concluded that the results of mindfulness and or exercise motivate them to continue using the techniques and exercises they were taught in the study. In the exercise group specifically, positive reinforcement and encouragement from the trainers motivated them to continue with their physical activity. The participants also wanted to see these interventions offered at the community level. Some suggested to use community centers, pre-existing programs such as OASIS or Silver Sneakers, or to offer it as continuing education. OASIS international in St. Louis, is a program for older adults that gives them opportunities to continue learning, volunteer, and improve health through activity. Silver Sneakers is a free fitness program for older adults to help encourage physical activity. One older adult suggested that the community should offer such interventions to all ages starting early in life. Other potential sites mentioned by the participants included churches, parks and libraries.

The major limitation of the current study is the fact that participation in the focus groups was voluntary, which could have resulted in selection bias. The older adults who chose to attend the focus groups may have been the most engaged and involved with the interventions. Nonetheless, this study confirms that older adults themselves find mindfulness and exercise to be helpful interventions. Another limitation to this study pertains to the responses form the individuals who said time commitment was a major barrier despite being retired. This implies that they were already social individuals. It would be important to know the effects of this intervention on individuals who do not have many social outlets in life.

## Conclusions

In summary, we showed older adults’ positive perceptions of mindfulness and physical training on the aging mind. Most participants reported mental, physical, and social improvements as a result of their intervention. This study is unique in that most investigations of MBSR and exercise in older adults do not collect qualitative data, and to date none have collected such data from a sample of individuals who have participated in both interventions, separately and combined, to compare perceptions across interventions.

## Supplementary information

**Additional file 1.** Non-structured Interview Guide. Non-structured interview guide used for the focus groups. 1. What were some of the benefits that you encounter while participating in the intervention? 2. What were some of the barriers that you encounter while participating in the intervention? 3. What were some of the facilitators that you encounter while participating in the intervention? 4. What are some of the recommendations for improvement in the intervention? 5. How to achieve higher engagement in the intervention? 6. What are some recommendations and personal strategies for maintenance of the practices learned during the intervention? 7. How could you continue the practices learned during this intervention (i.e. exercise, mindfulness) for the long-term? E.g. for the next 5+ years? 8. What is the feasibility of translating mindfulness and exercise interventions into community settings and how would this look like? Examples include: Self-management, technology delivered interventions, peer coach programs delivered by older adults, open gym alternatives such as fitness zones in public parks, and supervised and guided programs and other community engagement opportunities such as the Y and OASIS.

**Additional file 2.** Raw data from the focus groups classified by groups.

## Data Availability

The datasets used and/or analyzed during the current study are available from the corresponding author on reasonable request.

## Abbreviations

1-RM: One Repetition Maximum
MBSR: Mindfulness Based Stress Reduction
Min: Minutes
RCT: Randomized Control Trial
