# Supplementary material for: A qualitative study of older adults’ perspectives on initiating exercise and mindfulness practice
Source: BMC Geriatr. 2019 Dec 23;19:354. doi: 10.1186/s12877-019-1375-9 (PMC6927182; doi:10.1186/s12877-019-1375-9)
Supplement: Supplementary file 1 — Additional file 1. Non-structured Interview Guide. Non-structured interview guide used for the focus groups. 1. What were some of the benefits that you encounter while participating in the intervention? 2. What were some of the barriers that you encounter while participating in the intervention? 3. What were some of the facilitators that you encounter while participating in the intervention? 4. What are some of the recommendations for improvement in the intervention? 5. How to achieve higher engagement in the intervention? 6. What are some recommendations and personal strategies for maintenance of the practices learned during the intervention? 7. How could you continue the practices learned during this intervention (i.e. exercise, mindfulness) for the long-term? E.g. for the next 5+ years? 8. What is the feasibility of translating mindfulness and exercise interventions into community settings and how would this look like? Examples include: Self-management, technology delivered interventions, peer coach programs delivered by older adults, open gym alternatives such as fitness zones in public parks, and supervised and guided programs and other community engagement opportunities such as the Y and OASIS. [file 12877_2019_1375_MOESM1_ESM.docx]

- File name (Additional file 1)
- Title of data (Non-structured Interview Guide)
- Description of data: Non-structured interview guide used for the focus groups.

1. What were some of the benefits that you encounter while participating in the intervention?
2. What were some of the barriers that you encounter while participating in the intervention?
3. What were some of the facilitators that you encounter while participating in the intervention?
4. What are some of the recommendations for improvement in the intervention?
5. How to achieve higher engagement in the intervention?
6. What are some recommendations and personal strategies for maintenance of the practices learned during the intervention?
7. How could you continue the practices learned during this intervention (i.e. exercise, mindfulness) for the long-term? E.g. for the next 5+ years?
8. What is the feasibility of translating mindfulness and exercise interventions into community settings and how would this look like? Examples include: Self-management, technology delivered interventions, peer coach programs delivered by older adults, open gym alternatives such as fitness zones in public parks, and supervised and guided programs and other community engagement opportunities such as the Y and OASIS.
